# A rare case report of chylopericardium after esophageal cancer surgery: Diagnostic challenges and successful surgical management

**DOI:** 10.1016/j.ijscr.2025.111663

**Published:** 2025-07-15

**Authors:** ZePeng Zhao, Yu Zhu, Tao Zhang

**Affiliations:** a Jining Medical University School of Clinical Medicine (Affiliated Hospital), Jining 272000, Shandong Province, China; b Thoracic Surgery Department, Jining Medical University Affiliated Hospital, Jining 272000, Shandong Province, China

**Keywords:** Esophageal cancer, Chylous fistula, Chylopericardium, Low thoracic duct ligation, Surgery

## Abstract

**Introduction:**

Chylothorax is a rare but serious complication following thoracic surgery, characterized by the abnormal accumulation of chylous fluid within the pleural cavity or other intrathoracic spaces. Owing to its low incidence, chylothorax is often underrecognized by clinicians. Among its various forms, chylopericardium is even rarer and poses greater clinical challenges.

**Presentation of case:**

The patient was a 55-year-old woman who developed acute pericardial tamponade on postoperative day 7, presenting with sudden loss of consciousness and cardiorespiratory arrest. Pericardiocentesis revealed a large volume of chylous fluid. After 19 days in a coma, the patient gradually regained consciousness. On postoperative day 31, she underwent thoracoscopic low thoracic duct ligation and pericardial fenestration. Following the procedure, the volume of drainage decreased significantly, and she was subsequently discharged from the hospital.

**Discussion:**

Chylopericardium is a rare complication of esophageal cancer. It focuses on the causes of its occurrence and how to accurately diagnose and treat it.

**Conclusion:**

The abnormal occurrence of this case provides key insights into diagnosis, resuscitation, etiology speculation, surgical intervention, and patient rehabilitation, forming a mutually verified comprehensive cycle, which provides important guidance for the management of similar diseases.

## Highlights

• Reported cases of chyloperic effusion and cardiac tamponade after esophageal cancer surgery are extremely rare.
• Emergency puncture + low thoracic duct ligation + pericardial opening successfully saved the patient.
• Recommended preoperative oral olive oil to improve the rate of thoracic duct recognition

## 1 Introduction

Chylopericardium is an uncommon condition characterized by the aberrant accumulation of chyle in the pericardial cavity, typically resulting from rupture, obstruction, or impaired drainage of the thoracic duct. It is most frequently attributed to surgical injury of the thoracic duct, particularly during cardiothoracic operations [1]. Clinical manifestations are often nonspecific, including chest discomfort, dyspnea, palpitations, and fatigue; in severe cases, cardiac tamponade or cardiogenic shock may develop [2]. Diagnostic and therapeutic challenges arise from significant anatomical variability of the thoracic duct, making its precise localization difficult due to inter-individual differences, and thereby increasing the risk of intraoperative injury [3]; Additionally, preoperative fasting may reduce gastrointestinal contents and intraoperative complications, but also decreases lymphatic flow. Upon reintroducing diet postoperatively, chyle production may surge, elevating the risk of leakage [4,5]. The rarity of chylopericardium and the paucity of clinical documentation contribute to delayed diagnosis and complex management. Recently, video-assisted thoracoscopic surgery (VATS) has enabled more effective minimally invasive thoracic duct ligation, with reported success rates of 67–100 % [6,7]. This case provides valuable clinical insight into the treatment of chylopericardium, particularly when complicated by cardiogenic shock.

## 2 Methods

The work has been reported in line with the SCARE criteria [8].

## 3 Case presentation

Patient profile (General information): A 55-year-old woman with a history of hypertension was admitted with the chief complaint of “progressive dysphagia persisting for over four months.” Following comprehensive diagnostic evaluations, she underwent a successful radical esophagectomy.

The early postoperative course was relatively stable, with vital signs remaining within normal limits. However, the patient experienced persistent chest pain, which was managed with intramuscular injections of tramadol 100 mg twice daily. She also reported occasional episodes of chest tightness, most frequently occurring in the supine position. On postoperative day 1, in addition to routine intravenous nutrition, enteral nutrition was initiated at a daily volume of 500 ml, containing 8.5 g of fat.

**Fig. 1:**
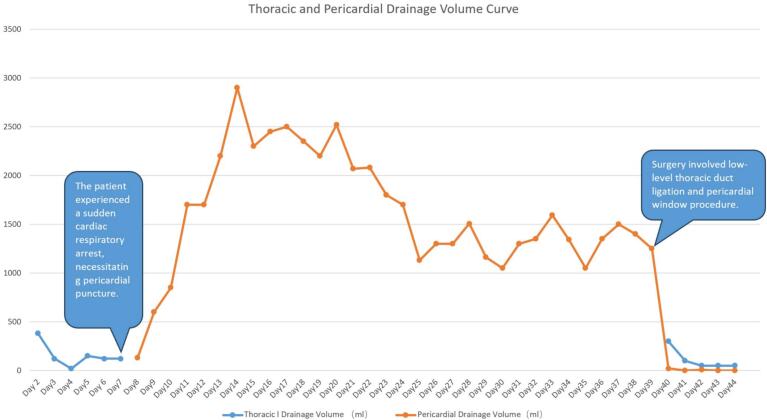
Postoperatively, the patient's drainage volume was initially within normal parameters. However, on the 7th day, she abruptly developed pericardial tamponade, compelling a pericardial puncture, which extracted a substantial quantity of turbid yellow fluid. The drainage volume reached its zenith, approximately 2900 ml, on the 14th day. Following cognitive recovery, the patient underwent thoracoscopic low-level thoracic duct ligation and pericardial window operation, leading to a marked decrement in drainage volume.

On postoperative day 7 at 08:00, the thoracic drainage tube was removed. At approximately 17:00 the same day, the patient developed acute cardiac distress immediately following defecation, which was rapidly followed by loss of consciousness and bilateral pupillary dilation. Considering a potential cardiac respiratory arrest, urgent external cardiac resuscitation, orotracheal intubation, and mechanical ventilatory assistance were administered. Bedside echocardiography revealed pericardial effusion (ranging from scant to moderate), necessitating a pericardial puncture, followed by sustained drainage through an inserted catheter, yielding a substantial volume of chylous fluid (Fig. 1 and Table 1).

**Table 1:** Summary of patient clinical data and surgical details.

Gender	Age	Diagnosis	Surgery type	Chylopericardium management			Surgical outcome	Prognosis
				Dietary restriction	Surgery	Thoracic adhesions		
Female	55	Esophageal cancer	Radical esophagectomy	√	√	×	Satisfactory	Cured
Drainage color	Chyle test (Sudan III staining)		Triglyceride in drainage fluid (mmol/l)	Cholesterol in drainage fluid (mmol/l)	Cholesterol/triglyceride ratio	Total drainage volume (ml)	Protein input (g)	Hospitalization duration (days)
Milky yellow/light red/light yellow	Mostly positive/weakly positive, few negative		0.6	0.31	1.9	51,650	590	44

The patient reacquired spontaneous respiration on the 16th day of the coma, exhibited gradual autonomous eye-opening, and accomplished simple command tasks by the 19th day. Consciousness gradually returned, and by the 29th day, she fully regained independent cognition and breathing. On the 31st day, she underwent thoracoscopic low-level thoracic duct ligation and a pericardial window operation. Postoperative drainage dramatically reduced from over 1000 ml to approximately 100 ml, facilitating the removal of pericardial and thoracic drainage tubes on the 4th day post-operation. The patient gradually recovered and was eventually discharged from the hospital.

## 4 Discussion

Chylothorax refers to the accumulation of chylous fluid within the pleural cavity, whereas chylopericardium—an even rarer entity—is defined by the abnormal retention of chyle in the pericardial space. Although both conditions are relatively uncommon in clinical practice, they can lead to significant circulatory or respiratory compromise, particularly in patients undergoing thoracic surgery, and thus warrant heightened clinical vigilance [1]. These complications typically present with non-specific early symptoms, such as mild chest pain, dyspnea, or fatigue, which can be easily mistaken for routine postoperative discomfort, often resulting in delayed or missed diagnoses [2]. In the present case, the patient's early postoperative vital signs were largely stable, and chest drainage output remained unremarkable, suggesting an initially smooth recovery. However, the patient persistently reported chest pain and developed posture-dependent symptoms, such as chest tightness when lying supine. Although these signs lack specificity, their persistence, coupled with the patient's subsequent rapid clinical deterioration, suggests that there may have been a slow accumulation of chylous fluid in the pericardial space postoperatively. As the condition progressed, the patient experienced sudden cardiopulmonary arrest. This clinical trajectory underscores the importance of considering anatomical variations of the thoracic duct and intraoperative risk factors, which may critically influence the timely recognition and management of chylous complications following thoracic procedures.

The thoracic duct is the largest lymphatic vessel in the human body, and is mainly responsible for draining lymph and chyle from the gastrointestinal tract and lower limbs to the venous system. It usually starts from the cisterna chyli, runs upward along the posterior mediastinum, and finally merges into the junction of the left subclavian vein and the internal jugular vein [3]. However, due to variations during embryonic development, the typical lymphatic drainage pathway only exists in about half of the population, and the anatomical structure of the thoracic duct may vary significantly. Studies have shown that the thoracic duct can run in a single, repeated, or plexiform pattern, and may cross the mediastinal midline in multiple thoracic segments. In rare cases, its terminal end may even open in atypical locations such as the azygos vein [9,10]. These anatomical variations will undoubtedly increase the difficulty of identifying and protecting the thoracic duct during surgery, leading to rare postoperative complications such as chylopericardium.

**Fig. 2:**
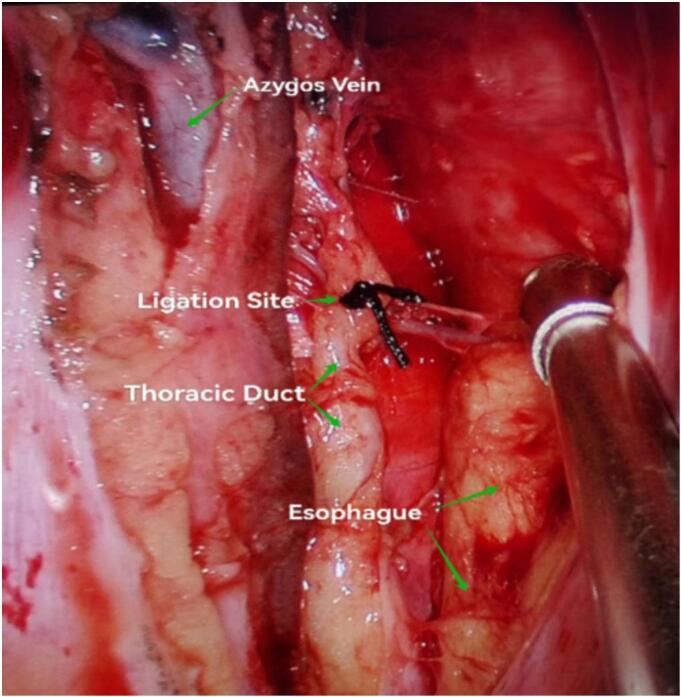
Low-level ligation of thoracic duct.

In this case, the azygos arch was injured during surgery and the surgeon ligated it, which may have led to the ligation of the thoracic duct by mistake or indicated that the patient had a rare anatomical variation in which the thoracic duct drained into the azygos vein or its arch. With the introduction of enteral nutrition, chyle continued to be produced and accumulated in the body, and the pressure in the thoracic duct gradually increased. During the postoperative period, the patient had difficulty defecating and strained many times, which significantly increased the intra-abdominal pressure, which may have induced the leakage or reflux of chyle into the pericardial cavity through potential lymphatic channels, eventually leading to pericardial tamponade and cardiac arrest. Although there is currently a lack of direct evidence to clearly prove that increased abdominal pressure can cause chyle to rupture and leak into the pericardium, previous studies have shown that when the thoracic duct pressure exceeds 15 cmH₂O, chyle can reflux and enter adjacent structures including the pericardium [1,11,12]. In addition, the patient's chest drainage volume decreased day by day after surgery, which seemed to be a smooth recovery on the surface, but in fact it may cover up the “hidden accumulation” process of chyle escaping into the pericardium in the early stage. Chylous fluid may have accumulated slowly since the early postoperative period until it reached the critical amount that triggered acute tamponade. This hypothesis is supported by the patient's delayed symptoms of pericardial tamponade and the typical chylous effusion elicited by puncture. Regardless of the mechanism, the patient's symptoms were rapidly relieved after pericardiocentesis, and the nature of the effusion further confirmed the diagnosis of chylopericardium. Thereafter, the patient underwent low thoracic duct ligation and pericardial fenestration (Fig. 2). The patient's symptoms improved significantly after the operation and he recovered and was discharged smoothly, which further verified the hypothesis of chylous reflux into the pericardial cavity from a clinical perspective.

**Fig. 3:**
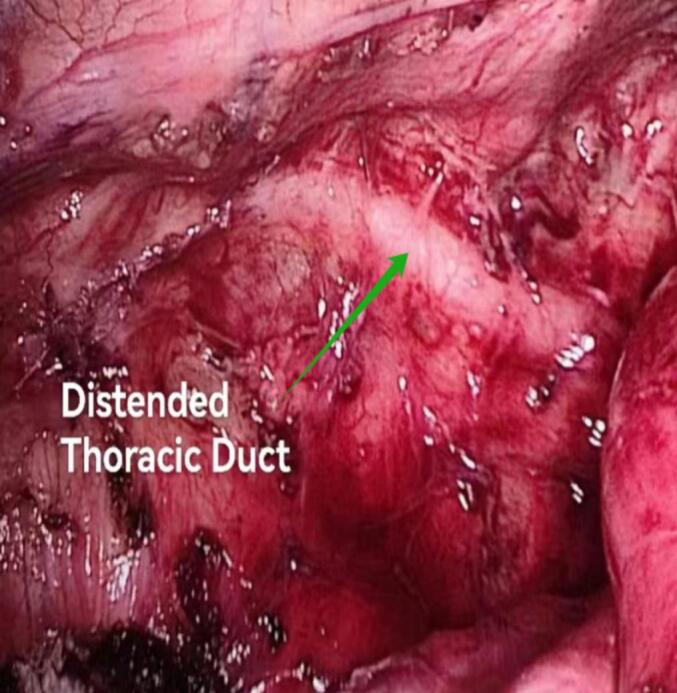
Thoracic duct distended after oral olive oil intake for 8 h.

Preoperative oral administration of olive oil has been shown to significantly enhance intraoperative visualization of the thoracic duct during esophagectomy. Yang et al. demonstrated that ingestion of 40 ml of olive oil 4 to 8 h before surgery facilitates thoracic duct distention and whitening, thereby reducing the risk of missed identification and unnecessary ligation of the duct [13]. However, considering the potential risk of aspiration during anesthesia induction, oral intake is recommended within 8 h prior to surgery (Fig. 3).

Chylorrhea fistulas following thoracic surgery are rare in clinical settings, with most patients achieving resolution through conservative approaches such as dietary restriction [14]. However, certain cases are more complex and require surgical management, typically involving thoracoscopic low-level ligation of the thoracic duct. A comprehensive understanding of the thoracic duct's anatomy and its potential variations is critical to minimizing iatrogenic injury. Acute chylopericardium leading to postoperative cardiogenic shock is an uncommon but severe complication, which can be effectively addressed through pericardial window creation and low-level thoracic duct ligation. The key therapeutic challenge lies in the prompt recognition and management of cardiac tamponade to prevent hemodynamic collapse.

## 5 Conclusion

Despite the low incidence of chylothorax following esophageal cancer surgery, surgeons must maintain a high level of vigilance intraoperatively to avoid thoracic duct injury and carefully assess any anatomical variations in the patient. Once chylothorax occurs, it should be diagnosed and treated in a timely manner. Conservative treatment is important, but for critically ill patients, active surgery rather than conservative treatment is the inevitable choice to save the patient's life. It has promotion value in clinical practice.

## CRediT authorship contribution statement

**Zepeng Zhao:** Writing - original draft, Writing - review & editing.
**Yu Zhu:** Writing – review.
**Tao Zhang:** Conceptualization, Writing-review & editing.

## Consent for publication

Written informed consent was obtained from the patient for publication and any accompanying images. A copy of the written consent is available for review by the Editor-in-Chief of this journal on request.

## Ethical approval

This study was approved by the Ethics Committee of the Affiliated Hospital of Jining Medical University under the approval number 2025-02-C011. All procedures involving human participants were conducted in accordance with the ethical standards of the institutional and/or national research committee and with the 1964 Helsinki Declaration and its later amendments or comparable ethical standards. Informed consent was obtained from all participants included in the study.

## Guarantor

Tao Zhang is the guarantor of this work and accepts full responsibility for the integrity of the content of this case report.

## Research registration number

1. Name of the registry: Not applicable
2. Unique identifying number or registration ID: Not applicable
3. Hyperlink to your specific registration: Not applicable.

## Funding

This study was supported by the Key R&D Program of Jining (2022YXNS077).

## Declaration of competing interest

The authors declare that they have no competing interests relevant to the content of this article.

## References

[1] Huang Z., Ge J. (2011). Current concensus on chylopericardium. Chin. J. Clin. Med..

[2] Khandaker M.H., Espinosa R.E., Nishimura R.A., Sinak L.J., Hayes S.N., Melduni R.M. (2010). Pericardial disease: diagnosis and management. Mayo Clin. Proc..

[3] Ilahi M., Lucia K. St, Ilahi T.B. (2025). Stat Pearls.

[4] Lv S., Wang Q., Zhao W., Han L., Wang Q., Batchu N. (2017). A review of the postoperative lymphatic leakage. Oncotarget.

[5] Yang Y.H., Park S.Y., Kim D.J. (2020). Chyle leakage after esophageal cancer surgery. Korean J Thorac Cardiovasc Surg..

[6] Bang J.H., Kim S.H., Park C.S., Park J.J., Yun T.J. (2015). Anatomic variability of the thoracic duct in pediatric patients with complex congenital heart disease. J. Thorac. Cardiovasc. Surg..

[7] Bender B., Murthy V., Chamberlain R.S. (2016). The changing management of chylothorax in the modern era. Eur. J. Cardiothorac. Surg..

[8] Kerwan A., Al-Jabir A., Mathew G., Sohrabi C., Rashid R., Franchi T. (2025). Revised Surgical CAse REport (SCARE) guideline: an update for the age of Artificial Intelligence. Prem. J. Sci..

[9] Johnson O.W., Chick J.F., Chauhan N.R., Fairchild A.H., Fan C.M., Stecker M.S. (2016). The thoracic duct: clinical importance, anatomic variation, imaging, and embolization. Eur. Radiol..

[10] Rabattu P.Y., Sole Cruz E., El Housseini N., El Housseini A., Bellier A., Verot P.L. (2021). Anatomical study of the thoracic duct and its clinical implications in thoracic and pediatric surgery, a 70 cases cadaveric study. Surg. Radiol. Anat..

[11] Nanjo S., Yamazaki J., Tsubuku M., Ohyama T., Ohtsuka T., Nakano H. (2004). Primary idiopathic chylopericardium: report of two cases. Ann. Nucl. Med..

[12] Zisis C., Rontogianni D., Charalambous E., Bellenis I. (2005). Lymphangiomatous hamartoma: cause or bystander of the isolated chylopericardium?. J. Thorac. Cardiovasc. Surg..

[13] Yang Y.B., Dai L., Wu Y.Y., Yan W.P., Liang Z., Lin Y. (2023). Pre-operative oil ingestion reduces the probability of thoracic duct trunk ligation during esophagectomy. Dis. Esophagus.

[14] Zhu X., Feng X., Huang Z., Xu W., Guo A., Xu J. (2024). Analysis of related factors and treatment effect of chylothorax after lung surgery. J. Thorac. Dis..

